# HPLC Quantitative Analysis of Rhein and Antidermatophytic Activity of *Cassia fistula* Pod Pulp Extracts of Various Storage Conditions

**DOI:** 10.1155/2013/821295

**Published:** 2013-09-19

**Authors:** Savita Chewchinda, Mansuang Wuthi-udomlert, Wandee Gritsanapan

**Affiliations:** ^1^Department of Pharmacognosy, Faculty of Pharmacy, Mahidol University, 447 Sri-Ayudthaya Road, Ratchathevi, Bangkok 10400, Thailand; ^2^Department of Microbiology, Faculty of Pharmacy, Mahidol University, 447 Sri-Ayudthaya Road, Ratchathevi, Bangkok 10400, Thailand

## Abstract

*Cassia fistula* is well known for its laxative and antifungal properties due to anthraquinone compounds in the pods. This study quantitatively analyzed rhein in the *C. fistula* pod pulp decoction extracts kept under various storage conditions using HPLC. The antifungal activity of the extracts and their hydrolyzed mixture was also evaluated against dermatophytes. The contents of rhein in all stored decoction extracts remained more than 95% (95.69–100.66%) of the initial amount (0.0823 ± 0.001% w/w). There was no significant change of the extracts kept in glass vials and in aluminum foil bags. The decoction extract of *C. fistula* pod pulp and its hydrolyzed mixture containing anthraquinone aglycones were tested against clinical strains of dermatophytes by broth microdilution technique. The results revealed good chemical and antifungal stabilities against dermatophytes of *C. fistula* pod pulp decoction extracts stored under various accelerated and real time storage conditions.

## 1. Introduction

*Cassia fistula* Linn. (Fabaceae) is native to southern Asia but now widely grown in tropical and subtropical areas as an ornament plant due to its beautiful, bright yellow flowers. Other vernacular names include golden shower, Indian larburnum, and pudding pine tree. *C. fistula*, a national tree of Thailand, is also known as ratchaphruek or khun in Thai. This plant is abundantly found in every region of the country as a flowering plant. The tree blooms profusely during summer, while the ripe pods are simply collected from the ground and thrown away as waste.

**Figure 1:**
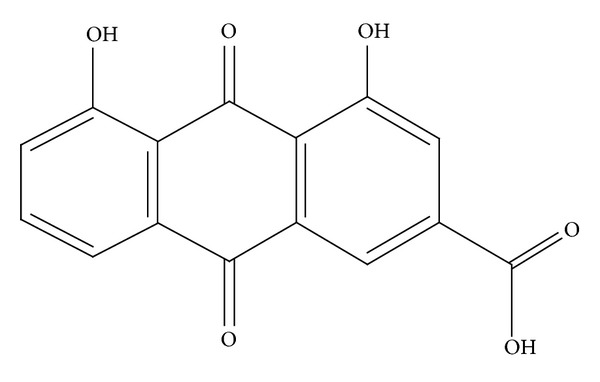
Chemical structure of rhein.

**Figure 2:**
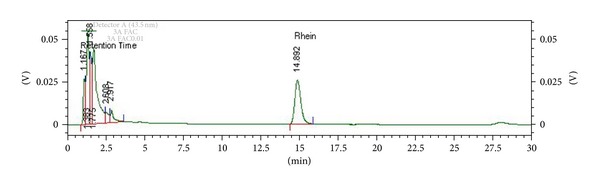
HPLC fingerprints of decoction extract from *C. fistula* pod pulp (at month 0).

*C. fistula* pod pulp is widely used in traditional medicines as a purgative/laxative drug and also used against various disorders such as skin diseases, diabetes, and other aliments [1, 2]. It possesses a mild, pleasant purgative action [3]. Various biological activities of the pod pulp such as antifungal, antibacterial, antioxidant, antileishmanial, antifertility, and hypolipidemic activities were reported [4–8]. The ripe pod pulp contains anthraquinones both in aglycone and glycosidic forms. The major anthraquinone in the pod of *C. fistula* is rhein (Figures 1 and 2). High concentrations of soluble sugars, volatile oils, and waxy and resinous substances are also found in the pod pulp [9, 10]. 

Dermatophytes are the most frequent pathogens that cause serious skin infection. The incidents have been increasing during last decades among immunocompromised patients such as organ transplant recipients, cancer, and HIV/AIDS patients [11]. Some* Cassia *species have been reported to treat fungal infection [12]*. C. alata, C. fistula, *and* C. tora* are recommended for primary health care in Thailand to treat ringworm and skin diseases. In Thai traditional medicine, laxative pills are obtained by boiling the ripe pod pulp of* C. fistula* with water. The pod pulp is also applied externally for skin eruption, ulcer, wound, eczema, and ringworm. From the previous study on extraction method for high content of anthraquinones from *C. fistula* pod, it revealed that the decoction with water according to Thai traditional use was found to be the appropriate extraction method [13]. Another work [14] reported high antifungal activity of anthraquinone aglycones extracted from glycosidic fraction of *Senna alata* leaves against clinical strains of dermatophytes. Therefore, it is interesting to determine and compare antidermatophytic activity of the decoction extract from *C. fistula* pod pulp and its hydrolyzed mixture that contained anthraquinone aglycones, to find a better antidermatophytic raw material from natural sources. The aims of this study were to determine rhein content in *C. fistula* pod pulp decoction extracts stored under real time storage and accelerated conditions and evaluate the antifungal activities against dermatophytes of these stored extracts and their hydrolyzed mixtures to indicate the chemical and biological stabilities of the extract from *C. fistula* pods. The contents of rhein in the extracts were analyzed using the validated HPLC method [15]. Antidermatophytic activities of the stored decoction extracts and their hydrolyzed mixtures were determined against *Trichophyton mentagrophytes*, *Trichophyton rubrum*, and *Microsporum gypseum.*

## 2. Materials and Methods

### 2.1. Plant Materials

The ripe pods of *C. fistula* were collected from Mahasarakham province in the northeastern part of Thailand in April 2010. They were identified by comparing with herbariums at The Forest Herbarium, Department of National Park, Wildlife and Plant Conservation, Ministry of Natural Resources and Environment, Bangkok. The voucher specimen (WCF0410) was deposited at Department of Pharmacognosy, Faculty of Pharmacy, Mahidol University. The ripe pods were cleaned with tap water and the pod pulp (without seed) was separated and kept in a tight container at 4°C until used.

### 2.2. Preparation of *C. fistula* Pod Pulp Extract

Fresh pulp of *C. fistula* ripe pod (20 g) was boiled with distilled water (200 mL) for one hour at 95–98°C, and the mixture was filtered. The extraction process was repeated until anthraquinones in the pulp were exhaustively extracted (tested by Borntrager's reaction). The filtrates were combined and evaporated to dryness on a boiling water bath to yield a decoction crude extract. Yield of the crude extract was recorded, and the extract ratio (weight of pod pulp : 1 g extract) was calculated.

### 2.3. Preparation of Extract Containing Anthraquinone Aglycones from *C. fistula* Pod Pulp Extract

The extraction process was modified from the assay procedure of hydroxyanthracene derivatives of *Cassia alata* described in the Standard of ASEAN Herbal Medicine, 1993 [16]. Ferric chloride hexahydrate solution of 10.5% w/v (20 mL) was added to the *C. fistula *pod pulp extract then refluxed for 20 min. The solution was added with concentrated HCl (1 mL) and refluxed for another 20 min then filtered after cooling down. The filtrate was shaken with 3 × 20 mL of chloroform in a separating funnel. The chloroform layer was combined and washed with 2 × 15 mL of distilled water, then evaporated to dryness to yield the hydrolyzed mixture containing anthraquinone aglycones.

### 2.4. Stability Studies of *C. fistula* Pod Pulp Extract

#### 2.4.1. Stability Study Condition

Three batches of *C. fistula* pod pulp decoction extract were kept in glass vials and aluminum foil bags at the accelerated (40°C ± 2°C/75% RH ± 5% RH) and real time storage conditions (30°C ± 2°C/75% RH ± 5% RH) for 6 months as described in ASEAN guideline on stability study of drug product, 2005 [17]. The samples were taken for quantitative analysis of rhein content and determination of their antifungal ability at 0, 3, and 6 months after storage.

#### 2.4.2. Chemical Stability Evaluation

The decoction extract of *C. fistula* pod pulp of each storage condition was analyzed for rhein content using HPLC [15]. The peak areas of rhein standard and samples indicated the content of rhein in each sample.

#### 2.4.3. HPLC Apparatus and Chromatographic Conditions

HPLC was performed on a Shimadzu Technologies modular model Class *VP *system consisting of a SCL-10A system, a UV-vis SPD-10A detector, LC-10 AD, and auto injector SIL-10A (Shimadzu, Japan). The analysis was carried out using a BDS Hypersil C18 column (250 × 4.6 mm, i.d. 5 *μ*m) (Thermo Fisher Scientific Inc., USA) with a BDS Hypersil C18 guard column (10 × 4 mm, i.d. 5 *μ*m) (Thermo Hypersil-Keystone, USA). The isocratic mobile phase was 0.5% aqueous acetic acid solution and methanol (40 : 60). The total running time was 30 min, and a flow rate was 1.0 mL/min. The UV detector monitored at 435 nm, while the injection volume was 20 *μ*L.

#### 2.4.4. Standard Solution Preparation

The rhein reference standard (Sigma, USA) was accurately weighed for preparing stock solution (1.0 mg/mL). Standard working solution of rhein was prepared by diluting the stock solution with 60% (v/v) methanol in the range of 1.25–20 *μ*g/mL.

#### 2.4.5. Sample Preparation

Each sample of *C. fistula* pod pulp decoction extract stored under various conditions was accurately weighed (0.1 g), dissolved in 60% (v/v) methanol, and adjusted to 10 mL in a volumetric flask. The solution was filtered through a 0.45 *μ*m nylon membrane filter and analyzed in triplicate.

### 2.5. Antifungal Assay

#### 2.5.1. Inoculum Preparation

Clinical isolated strains of* T. mentagrophytes*, *T. rubrum*, and *M. gypseum *were used in this experiment. Clinical strains identification were based on macroscopic and microscopic examination of cultures. Prior to testing, the strains were separately inoculated on Sabouraud dextrose agar plates and incubated at 28°C for 7 days to ensure the viability and purity of the inoculum. *T. mentagrophytes* DMST 19735 (DMST: Department of Medical Sciences Type culture collection, Thailand) was included as a reference strain.

#### 2.5.2. Determination of Minimum Inhibitory Concentration

Antifungal activities of rhein standard (Sigma, USA), decoction extracts of *C. fistula* pod pulp stored under each storage condition, and their hydrolyzed mixture containing anthraquinone aglycones were tested by broth microdilution assay for antifungal susceptibility in accordance with the Clinical and Laboratory Standard Institute guidelines in the M38-A document on filamentous fungi [18] with modified incubation temperature. Briefly, in U-shaped 96 wells plate, each well was filled with 100 *μ*L of the twofold serially diluted test samples and 100 *μ*L of the fungal spore suspension to obtain final concentration of inoculum at 1 to 3 × 10^3^ CFU/mL. Growth control was filled with 100 *μ*L of the inoculum and 100 *μ*L of RPMI 1640 medium. Each sample was tested in duplicate, while ketoconazole was used as a positive control. The MICs, the lowest concentration of the sample which shows no visible growth after incubation at 28°C for 4 days, of rhein, decoction extracts, and their hydrolyzed mixtures containing anthraquinone aglycones while the MICs of ketoconazole were defined as approximately 80% of inhibition when compared to growth control results.

## 3. Results and Discussion

According to the evaluation criteria of ASEAN guideline on stability study of drug product, 2005, significant change is defined as more than 5% difference from its initial value. Rhein contents in the decoction extracts of *C. fistula* pod pulp kept under various storage conditions determined by HPLC were found in the range of 0.0788 ± 0.005 to 0.0823 ± 0.001% w/w (Table 1).

The rhein contents remained more than 95% when compared with the initial amount. The results showed no significant change of the extracts kept in glass vials and in aluminum foil bags for 6 months, and the acceptance criteria were met. This indicates good chemical stability of the *C. fistula* pod pulp decoction extract. Storage of the decoction extract in glass vials or in aluminum foil bags promoted no difference on the chemical stability of the extract.

For antifungal assay, many reports [19–21] showed no difference on the MIC of isolates incubated at 28 and 35°C. Moreover, dermatophyte strains show optimal growth between 4 and 15 days of incubation at 28–30°C [22]. Thus, the incubation temperature for the broth microdilution assay of antifungal susceptibility testing in this study was performed at 28°C instead of 35°C. 

The decoction extracts of* C. fistula* pod pulp kept under various storage conditions showed very low antifungal activity (MIC > 1,000 *μ*g/mL). The MICs of hydrolyzed mixtures and rhein standard are shown in Table 1. Anthraquinone aglycones in the hydrolyzed mixtures could inhibit the growth of *T. mentagrophytes* DMST 19735, *T. mentagrophytes* and *M. gypseum* at the same MIC of 500 *μ*g/mL, and *T. rubrum *at MIC of 250 *μ*g/mL. Whereas rhein standard showed better activities against all tested dermatophytes at the same MIC of 125 *μ*g/mL. For the drug control, ketoconazole, its inhibitions against *T. mentagrophytes* DMST 19735*, T. mentagrophytes*, *T. rubrum, *and *M. gypseum* were found at MICs of 2, 1, 1, and 2 *μ*g/mL, respectively. Our results on antifungal activity of the hydrolyzed extracts containing anthraquinone aglycones from *C. fistula* pod pulp were correlated with the previous study of Duraipandiyan and Ignacimuthu 2010 [23] that rhein from *C. fistula* flower exhibited good activity against dermatophytes. Also, the data confirmed the report on *S. alata* leaves that anthraquinone aglycones promoted better antidermatophytic activity than anthraquinone glycosides [14]. This work supported the traditional use of *C. fistula* pods as a drug for treatment of fungal skin diseases.

## 4. Conclusions

The decoction extract of *C. fistula* pod pulp, which contains high amount of soluble sugars, showed very low antidermatophytic activity but its hydrolyzed mixture containing anthraquinone aglycones was active against all dermatophytes:* T. mentagrophytes* DMST 19735, *T. mentagrophytes*, *T. rubrum, *and *M. gypseum*. The decoction extract of* C. fistula* pod pulp was chemically stable after 6 months of storage under the accelerated and real time storage conditions. This could be implied that *C. fistula* pod pulp decoction extract should enable a tentative shelf life of 24 months [24]. Glass vials and aluminum foil bags promoted no difference on the chemical and antifungal stabilities of the pod pulp extracts. The hydrolyzed pod pulp mixture with anthraquinone aglycones should be selected for fungal treatment. Anyhow, chemical and antifungal stabilities of the stored hydrolyzed extracts under various storage conditions should be further studied to be assured.

## Figures and Tables

**Table 1:** The content of rhein in the decoction extract and antifungal activity against dermatophytes of the decoction extract and its hydrolyzed mixture containing anthraquinone aglycones of *C. fistula* pod pulp kept under various storage conditions of stability studies.

Time (month)	Packaging	Storage condition	Rhein content^a^ (% w/w)	*T. mentagrophytes* DMST 19735 and *T. mentagrophytes* MIC (*µ*g/mL)		*T. rubrum* MIC (*µ*g/mL)		*M. gypseum* MIC (*µ*g/mL)	
				Decoction extract	Hydrolyzed mixture	Decoction extract	Hydrolyzed mixture	Decoction extract	Hydrolyzed mixture
0			0.0823 ± 0.001 (100.00%)^b^	>1000	500	>1000	250	>1000	500

3	Glass vial	Real time	0.0829 ± 0.001 (100.66%)	>1000	500	>1000	250	>1000	500
	Aluminum foil	Real time	0.0815 ± 0.001 (98.96%)	>1000	500	>1000	250	>1000	500
	Glass vial	Accelerated	0.0817 ± 0.003 (99.25%)	>1000	500	>1000	250	>1000	500
	Aluminum foil	Accelerated	0.0809 ± 0.002 (98.23%)	>1000	500	>1000	250	>1000	500

6	Glass vial	Real time	0.0791 ± 0.002 (96.11%)	>1000	500	>1000	250	>1000	500
	Aluminum foil	Real time	0.0794 ± 0.001 (96.46%)	>1000	500	>1000	250	>1000	500
	Glass vial	Accelerated	0.0790 ± 0.001 (95.95%)	>1000	500	>1000	250	>1000	500
	Aluminum foil	Accelerated	0.0788 ± 0.005 (95.69%)	>1000	500	>1000	250	>1000	500

^a^Expressed as mean ± SD (*n* = 3) and ^b^content of rhein in *C. fistula* decoction extract calculated as 100% at day 0. Real time = 30°C ± 2°C/75% RH ± 5% RH. Accelerated = 40°C ± 2°C/75% RH ± 5% RH.
